# The feasibility of delivering constraint-induced language therapy via the Internet

**DOI:** 10.1177/2055207617718767

**Published:** 2017-07-02

**Authors:** Rachelle Pitt, Deborah Theodoros, Anne J Hill, Amy D Rodriguez, Trevor Russell

**Affiliations:** 1 Centre for Research in Telerehabilitation, University of Queensland, Australia; 2 School of Health and Rehabilitation Sciences, University of Queensland, Australia; 3 Center for Visual and Neurocognitive Rehabilitation, Atlanta VA Medical Center, USA

**Keywords:** Telerehabilitation, aphasia rehabilitation, constraint-induced language therapy

## Abstract

**Objective:**

This study is designed to determine the feasibility of the provision of an evidenced-based aphasia therapy, constraint-induced language therapy, via telerehabilitation. It describes the computer software that was developed specifically for the delivery of constraint-induced language therapy in the online environment and presents two case studies.

**Methods:**

Two participants with chronic aphasia were assessed before and after a two-week intensive treatment block of constraint-induced language therapy delivered via Web-based videoconferencing. The group-based, dual card request game utilized in face-to-face constraint-induced language therapy was transformed into an innovative and user-friendly Web-based game – Internet constraint-induced language therapy (iCILT). Participants accessed iCILT via the Internet from their own home every weekday for two weeks. Language and quality of life measures were administered pre- and post-therapy in order to detect treatment effects. Participant satisfaction information was also recorded.

**Results:**

Online delivery of iCILT was technically feasible and participant satisfaction was high with a number of benefits associated with telerehabilitation identified by participants. Post-treatment performance for language functioning and communication-related quality of life was variable for each participant, however improvements in naming were noted.

**Conclusions:**

Constraint-induced language therapy delivered via telerehabilitation may be a feasible alternative to traditional face-to-face therapy for people with chronic aphasia.

## Introduction

Aphasia is an acquired neurogenic language disorder primarily caused by stroke.^1^ It is recognized as a chronic condition in which speaking, listening, reading, and writing may be impaired.^2^ Aphasia affects the opportunities for communication and the quality of communication exchanges,^3^ restricts participation in activities of daily living,^4^ and negatively impacts on psychosocial well-being and quality of life.^5^

Despite these significant impacts, people with aphasia often have difficulty accessing evidence-based speech-language pathology services.^6^ A number of factors have contributed to this trend including reductions in available services as a result of rising healthcare costs and funding limitations,^6–8^ limited access to services in rural or remote locations,^9^ and increased demand for services as a result of the ageing population.^6,10^ Further, co-occurring mobility and/or cognitive difficulties are common, with motor impairment evident in 80% of individuals post-stroke,^11^ and up to 78% of patients exhibiting some cognitive difficulties.^12^ These co-morbidities may result in limitations that further prevent access to speech-language pathology services outside of the home.^8,13,14^

Providing optimal services to individuals with aphasia requires clinicians to seek treatment delivery approaches that are “outside the box” (p.39).^15^ Telerehabilitation may be one such approach as it reduces travel, improves the timeliness of services, and allows for increased intensity of services to be provided.^16–19^ Therefore, delivery of therapy via telerehabilitation has the potential to facilitate access to services and decrease costs associated with providing optimal care for people with aphasia.^9,18,20^

Studies investigating treatment of aphasia via telerehabilitation have consistently found benefits such as improvements in language functioning, reduced travel time, reduced costs for the rehabilitation service, and increased intensity of services.^19,21–24^ Unfortunately, only the study conducted by Cherney and colleagues^24^ describes the therapy provided and evidence supporting the efficacy of the intervention in sufficient detail for this to be replicated. Although the studies reported here provide some support for the telerehabilitation application of aphasia treatments, it is necessary to identify treatments with previously established efficacy and investigate their effect in the context of an online environment. Further, the majority of research to date has considered asynchronous (not in real time) therapy delivery where the participant is provided therapy activities to complete independently with the clinician monitoring performance remotely. Although there are a number of benefits associated with this mode of service delivery, determining the feasibility of real-time aphasia therapy via telerehabilitation is essential for establishing evidence for treatment across the continuum of care.

One therapy approach that may be appropriate for delivery in the online environment is constraint-induced language therapy (CILT). First described by Pulvermuller et al.,^25^ the approach has been shown to yield substantial and stable improvements in language function for people with chronic aphasia that carry over to everyday life.^25–31^ CILT incorporates three critical neuroscientific principles including:

1. Massed practice: it is beneficial to have more intensive practice (more therapy hours) at an increased frequency (number of therapy hours per time).
2. Behavioral and communicative relevance: it is beneficial to practice language in relevant contexts that exploit non-linguistic actions and object perceptions.
3. Focusing: therapy that guides patients to use spoken language as the main route of communication increases stimulation opportunities for impaired language functions and overcomes learned non-use.^32^

These principles are realized through the intensive delivery of language action games that allow for the practice of speech acts in communicatively relevant contexts. These games utilize pre-determined object picture cards in games that require both non-communicative and verbal communicative actions. CILT typically involves two to three people with aphasia who communicate and compete with each other. A trained clinician, usually a speech-language pathologist (SLP), provides cuing and guidance to produce targets correctly and determines the complexity of the spoken utterances in each turn. As CILT relies primarily on verbal communication and uses a pre-determined set of treatment stimuli, it may be highly adaptable to real-time online administration. However, it is recognized that the interactions between participants, the clinician, and the object picture cards may be different in an online environment. Further, the perceptual, cognitive, and psychomotor capacity of patients with aphasia and stroke may present unique barriers to using the technology required to access online CILT.^33,34^ Therefore the primary aim of this study was to investigate the feasibility of delivering high-intensity, evidenced-based CILT via telerehabilitation. A secondary aim was to describe participant responses to the therapy provided.

## Materials and methods

### Participants

Two participants with chronic aphasia were recruited to the study through the University of Queensland Aphasia Registry. LR, a 41-year-old male, had experienced an ischemic stroke secondary to a bacterial infection 24 months prior to the study. He presented with acquired aphasia characterized by moderate-severe word finding difficulties as evidenced by performance on the Comprehensive Aphasia Test (CAT).^35^ Clinical observation of connected discourse suggested mild-moderate apraxia of speech. LR was living at home with his wife who agreed to provide technology support when needed. Although LR was able to independently access therapy services, access to face-to-face CILT therapy would have required him to travel 25 km every day for two weeks to the clinic, and locate and pay for parking at the location. LR had received CILT face-to-face one year prior to the study as part of an intensive comprehensive aphasia program. BG, a 78-year-old female, suffered an ischemic stroke 23 months prior to the study. BG’s assessment results on the CAT revealed more severe acquired aphasia with severe word-finding difficulties and moderate-severe comprehension difficulties. BG lived alone but received technology support during all sessions from family members. BG was unable to drive and would have had to rely on working family members for transport to therapy sessions. BG had not received CILT prior to this study.

### Procedure

Prior to commencing the study, ethical clearance was obtained through the University of Queensland Behavioural and Social Sciences Ethical Review Committee. Participants were assessed face-to-face by an independent assessor who was a qualified SLP immediately before and after treatment. Language function was assessed across a range of areas including comprehension of spoken and written language, naming, repetition, reading and writing using the CAT.^35^ The impact of aphasia on everyday life was measured using the Assessment for Living with Aphasia (ALA)^36^ which provides information regarding the impact of the communication impairment across a number of domains including language impairment, participation in everyday life, communication environment, and personal factors.

Participant satisfaction with the online treatment was measured using an aphasia-friendly satisfaction questionnaire at the completion of the treatment block. The questionnaire comprised 16 questions exploring the participant’s perception of the benefits of therapy, video and audio quality, efficiency and ease of interaction with the SLP, and overall satisfaction. Participants indicated their responses on a five-point rating scale provided in an aphasia-friendly format with clear text and pictures. Participants were also able to provide feedback regarding positives and negatives of the therapy in response to two open-ended questions.

Technical feasibility was monitored by the treating SLP who kept a log of network connection, audio and video quality, and performance of the Internet constraint-induced language therapy (iCILT) software. Reflective notes on providing therapy online were also recorded by the treating SLP.

### Treatment procedures

#### Treatment environment

Participants accessed therapy from their own homes via a high speed broadband Internet connection (Asymmetric Digital Subscriber Line (ADSL)). Participants used their own desktop computers however were provided a Logitech c270 HD 3MP webcam and Plantronics Audio 478 USB Stereo headset microphone for the duration of the study. The treating clinician was a qualified SLP with experience in aphasia rehabilitation and telerehabilitation. The Web-based dual card game developed for this project (iCILT), and the videoconferencing software (Adobe Connect, Adobe Systems Software Ireland Ltd, Ireland) were simultaneously run through an HTML 5 Internet browser. Adobe Connect was hosted on the University of Queensland network and allowed simultaneous video and audio between both participants and the SLP. The webcam was positioned so that participants could only see the face of the other player in order to minimize the effectiveness of gesture in conveying a response. In this way, the interaction emulated the barrier approach used in face-to-face CILT interventions^27^ whilst still allowing eye contact and facial expression to be viewed by all. This was considered important for building rapport between participants and the SLP,^37^ observation of SLP cues by participants, and ensuring participants could view naturally occurring social feedback when targets were successfully produced.^29^ To access both Adobe Connect and iCILT Web pages, shortcuts were created on the desktop of the participants’ computer at the initial assessment session conducted at their home. Participants were only required to click on the shortcuts and enter their name to access the therapy sessions. At the initial assessment session, LR was provided with training and practice until he could independently access the Web pages. BG’s family members were also trained over the phone prior to treatment commencing as she did not feel confident in accessing the online treatment. Aphasia-friendly written information describing the steps required to establish the connection was also provided to both participants at the initial assessment session. Due to the complex nature of aphasia and the possibility of co-occurring higher level cognitive disturbance, the provision of adequate training and aphasia-friendly materials and communication with family support was considered essential to ensure that the participants were able to access the online treatment.

#### Therapy schedule

Both participants were treated concurrently by one SLP over the Internet and had access to three hours of therapy per day, five days per week for two weeks as per the face-to-face protocol. Neither participant received the full 30 h of therapy due to a combination of fatigue and network connection difficulties. If one participant was unable to continue in the session, treatment was continued for the remaining participant with the clinician acting as the second player in iCILT as well as providing cues and feedback. The number of participant turns in the dual card game was recorded for each participant in each session in order to determine the number of attempts at a target and the treatment intensity. The information regarding the session details is presented in Table 1.

**Table 1.** Internet constraint-induced language therapy (iCILT) session information.

	Participant	
	LR	BG
Mean (SD) session length, min	164.9 (12.05)	151.6 (14.8)
	Range = 143–179	Range = 128–172
Total therapy, h	27.48	25.27
Mean (SD) number of turns per session	76.4 (9.41)	63.5 (10.6)
	Range = 62–89	Range = 48–85
Number of therapy hours missed due to fatigue	1.63	3.85
Number of therapy hours missed due to network connection difficulties	0.89	0.89

SD: standard deviation.

#### iCILT

**Figure 1.**
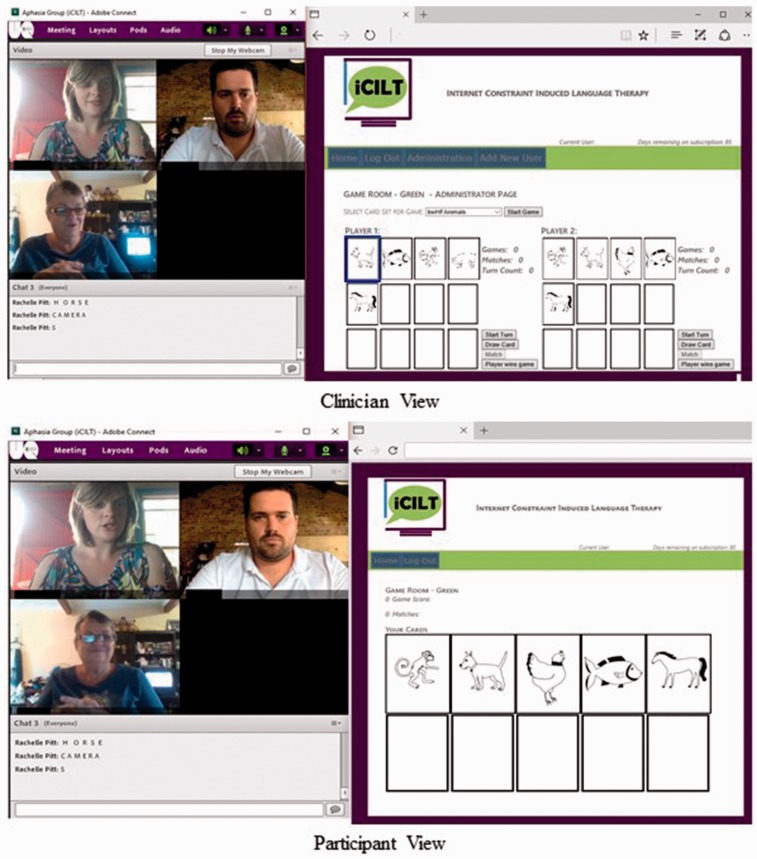
Internet constraint-induced language therapy (iCILT) screenshots.

The online treatment tool, iCILT, was specifically developed by the research team for the delivery of CILT via telerehabilitation. The key principles of face-to-face CILT are intensive practice, behavioral relevance, and constraint of non-verbal communication through focusing.^32^ iCILT maintained these principles in that it replicated as closely as possible the dual card request language action game first described by Pulvermuller et al. ^25^ with two participants with aphasia interacting with each other, and followed the treatment task sequences and levels of difficulty described by Maher et al.^27^ In the online delivery, participants entered the game screen in which there were spaces for 10 “cards” to be dealt. The cards dealt contained line drawings in either black and white or color and depicted high or low frequency words from one of five semantic categories: everyday items, around the house, animals, clothes, and food. The clinician initially dealt five cards to the participants with the category selected by the clinician at random and the level of difficulty such as color or word frequency determined according to participant performance. The cards from the selected category were randomly dealt by the Web game and appeared on the participants’ screens (see Figure 1). The clinician was able to view both participants’ card sets in order to provide appropriate cues when it was their turn to request. At the start of a turn, the participant clicked on a card they wished to request which highlighted the card on both their screen and on the clinician’s screen. Highlighting a card in this way allowed the clinician to facilitate accurate production of the request and reduce errors by providing either semantic or phonological cues or modeling. This also required the participant to interact with the picture card to take advantage of the benefits of non-communicative behavioral relevance. The complexity of the request was determined by the participants’ performance and gradually shaped from single words to complex utterances. The other participant was required to provide an appropriate response indicating if they did or did not have a matching card. These request and response sequences are key to maintaining the language action contexts of CILT. When there was a match, the clinician cleared the matched cards from the participant’s screen which incremented a match counter and the game continued. Alternatively, if participants had adequate dexterity and understanding of using the computer, they could highlight the matching picture card on their screen and the cards would be cleared from their deck. Again, this aimed to maintain some of the non-communicative motor actions involved in the language action game. When there was no match, the clinician dealt the player a new card and their turn ended. Figure 1 shows the screen view from both the participant and clinician perspectives.

### Data analysis

The raw scores for LR and BG on the CAT were converted to standardized T-scores according to the CAT test manual. The difference in T-scores from baseline to post-treatment was calculated and using the test–retest reliability data from the test manual, it was possible to determine the amount of change that would be required to be clinically significant. The responses obtained on the ALA and participant satisfaction questionnaire and researcher technology log were analyzed descriptively.

## Results

### Technical feasibility

A review of the technology log kept by the treating clinician revealed a number of findings relating to connection to Adobe Connect, video and audio connection, and use of iCILT. The most significant connection issue involved video and audio disconnection with LR losing video on 25 occasions and audio on 10 occasions during the 10-session treatment block. The number of disconnections varied per session with three sessions having no connection issues and the remaining seven sessions having up to five disconnections of video and/or audio. BG lost video on three occasions in separate sessions and audio on two occasions in one session. These issues were usually resolved by reconnecting audio and video or exiting the meeting room and rejoining. For two sessions however, connecting LR’s video continually resulted in loss of audio and the session was completed with no video streaming from his end. Another issue identified with Adobe Connect was difficulty connecting to the meeting room. On one occasion each during the 10-session treatment block, both participants were unable to join at the initial attempt. This was resolved with a phone call to participants and full restart of their computer. LR lost connection to the meeting room during the session on nine occasions and the clinician was lost connection on four occasions. Both LR and the clinician were able to reenter the Adobe Connect room after refreshing the browser.

The software game iCILT successfully allowed for the delivery of the “Go Fish” request game according to the clinician log. Both participants and the clinician were able to enter the game room on all occasions with no difficulties. No significant errors with the program were recorded. Both participants were able to use the software independently during gameplay, although BG needed assistance from family at the initial log in. The clinician was satisfied with the usability of iCILT and was easily able to control the game including dealing cards, viewing and managing participants’ card selections and matching pairs. The clinician noted that although semantic and phonological cues were provided to participants as needed, delays in video and audio through Adobe Connect negatively impacted the timing of these cues on some occasions. The clinician noted that the appropriate progression of the participants through the hierarchy of difficulty according to the color and frequency of the cards dealt was also possible using the iCILT software and appropriately followed participant performance in therapy sessions. As such, the delivery of CILT using the iCILT software replicated the sequence of events followed in the face-to-face environment.

### Participant satisfaction

The results from the satisfaction survey are presented in Table 2. Participant satisfaction was very high with both participants responding in the neutral or positive range for all questions. Both participants reported that iCILT had benefited them with respect to communication and saving money. High satisfaction with the technology was also noted, with both participants reporting they could see and hear the clinician easily and that the interaction went smoothly. Participants differed in the assistance needed to access the therapy online. BG reported definitely needing help and LR reported that no help was required. Written comments revealed that LR felt it would be better to match participants in the sessions according to aphasia severity more closely, and that using a greater variety of tasks would improve the treatment. However overall, both participants were satisfied with the iCILT treatment.

**Table 2.** Participant satisfaction questionnaire responses.

	No definitely not	No I don’t think so	Neutral	Yes I think so	Yes definitely so
Has your communication improved since being in iCILT?				• ⋄	
Have you gained new skills from participating in iCILT?			•		⋄
Could you easily see the speech pathologist?				•	⋄
Could you easily hear the speech pathologist?				•	⋄
Did you feel comfortable receiving treatment online?				•⋄	⋄
Did you need help using the computer?		•			⋄
Did you find having therapy at home was easier?				•	⋄
Do you think therapy online is a good way to receive treatment?			•		⋄
Did you save travel time during iCILT?			⋄	•	LR • BG ⋄
Did you save money during iCILT?				•	⋄
Would you have online treatment again?			⋄	•	
Would you prefer to have face to face therapy?		•	⋄		
Did iCILT run smoothly?				•	⋄
Was iCILT as good as you thought it would be?				•⋄	
Would you recommend iCILT to others?			•		⋄
Overall, were you satisfied with the iCILT program?				•	⋄

i CILT: Internet constraint-induced language therapy.

LR: •; BG: ⋄.

### Therapeutic effect

Table 3 reports the results of language and communication quality of life (QOL) measures for LR and BG.

**Table 3.** Participant pre- and post-assessment results.

Assessment	LR pre	LR post	Difference	BG pre	BG post	Difference
CAT T-score						
Spoken comprehension	60	62	2	46	46	0
Written comprehension	60	**73**	**13** ^a^	44	45	1
Repetition	49	49	0	44	45	1
Naming	59	**68**	**9** ^a^	42	44	2
Spoken pic description	53	52	–1	46	46	0
Reading	49	49	0	38	38	0
Writing	57	59	2	41	41	0
Written pic description	55	59	4	42	42	0
ALA average						
Aphasia	2.1	2.1	0	1.50	3	1.5
Participation	2.74	2.62	–0.12	3.21	3.29	0.08
Environment	3	2.50	–0.50	2.86	2.63	–0.23
Personal	3.45	2.91	–0.54	3.41	3.59	0.18
Life with aphasia	2	2	0	3.50	4	0.50
Total	2.87	2.61	–0.26	3.01	3.29	0.28

ALA: Assessment for Living with Aphasia; CAT: Comprehensive Aphasia Test.

a Difference between pre-test and post-test score clinically significant according to CAT manual test–retest minimum change.

LR demonstrated a clinically significant improvement on the subtests of Naming and Written Comprehension on the CAT assessment. This suggests improved word retrieval for confrontation naming of objects and improved ability to gain meaning from written information. BG demonstrated minimal changes in performance on the CAT with an increase in 1–2 points following treatment for Naming, Written Comprehension, and Repetition.

LR’s ratings in the domains of Aphasia and Life with Aphasia did not change, and ratings in the domains of Participation, Environment, and Personal decreased (total score 2.87 pre-treatment and 2.61 post-treatment). As such, treatment did not result in positive changes to communication-related QOL for LR. Small increases in ratings were noted across all domains on the ALA for BG (see Table 3).

## Discussion

This study demonstrated that the delivery of an intensive aphasia treatment via telerehabilitation (iCILT) was feasible and viewed favorably by recipients. The results from these two case studies suggested that iCILT yielded some improvements in language functioning although it did not result in changes in communication-related QOL.

### Feasibility of telerehabilitation delivery of CILT

Although a number of issues were noted with the videoconferencing software Adobe Connect, high participant satisfaction and the functionality of iCILT suggest that delivery of CILT via telerehabilitation is feasible. The most significant issue noted during treatment was the connection issues experienced in Adobe Connect. These issues may have been related to the demand on the university server through which Adobe Connect was run and may be resolved if future sessions are hosted on an independent server. Consideration of an alternative videoconferencing platform for this treatment may also be an option. Despite these issues both participants indicated that they could hear and see the clinician clearly and that they felt the treatment ran smoothly. This finding is consistent with previous research conducted by Theodoros et al.^38^ who found participants remained satisfied with telerehabilitation even when difficulties with technology were noted. The authors suggested that participants may respond in this way due to the novelty of the online therapy. It is also possible that the participants were providing feedback on the iCILT card game as opposed to the Adobe Connect application. The Web-based dual card game was purposefully designed to maintain the key features of CILT whilst ensuring the program was as user-friendly as possible. The screen displays were simple and easy to navigate and accessing the treatment site involved the participants opening one link from the desktop and entering their name to sign into the game screen. One participant (LR) was able to access all treatment sessions without difficulty as indicated on the satisfaction questionnaire. However, BG required assistance from a family member at each session: her more severe receptive difficulties may have influenced her ability to understand the instructions for accessing therapy. A previous study investigating telerehabilitation assessment of people with aphasia indicated that severity level did not impact on participant satisfaction.^39^ However, that study was conducted in a clinical setting and participants were not required to turn the system on, enter log in details, or connect video and audio. Both participants reported using computers prior to the study, with LR reporting independently engaging in a number of computer-based tasks on a daily basis: for example email, social networking, viewing and editing digital photos, and Web surfing. BG’s computer use post-stroke, however, was primarily restricted to email and viewing photos with family support. Therefore the differing experience in computer use prior to the study may have influenced the participants’ ability to independently access the treatment. It is promising that the clinician log identified that both participants were able to independently use iCILT during treatment following initial assistance to log in. Future studies should focus on improving connectivity and ease of access to the videoconferencing software through additional training and support materials.

Both participants reported that receiving therapy online was easier than accessing face-to-face therapy and allowed them to save travel time or money. Although LR was able to drive, travelling to and from therapy would have been time consuming and costly, and family commitments restricted access to a vehicle for everyday use. BG was totally dependent on her family for transport to therapy. As these family members also had work and personal commitments, it is unlikely that BG would have been able to access the same intensity of therapy outside of the home. The unique benefits of telerehabilitation experienced by participants in this study are well recognized in the literature and are also associated with the reduced need for the clinician to travel and reduced costs to the rehabilitation facility.^40^ Reduced travel and costs may allow services to improve the timeliness of therapy provided and increase the intensity of services provided.^19,41^ In the case of CILT therapy, the delivery of high-intensity services in a timely and cost effective manner is particularly important as intensity is recognized as a critical factor in the efficacy of this treatment. Further, commonly cited barriers to the implementation of traditional CILT relate to the difficulty of people with aphasia accessing the intensity of the therapy that is necessary to effect change in language function.^25,27,32^ Transport to and from the rehabilitation facility to access face-to-face CILT has also been cited as a barrier to accessing treatment.^27^ Therefore iCILT may make access to high-intensity treatment a reality for people with aphasia who have reduced mobility, limited transport options, or who are experiencing financial difficulties.

Overall both participants’ responses to all questions on the survey were neutral or positive and they were both satisfied with the online treatment. Such positive responses are consistent with previous literature in relation to speech or language services delivered via telerehabilitation,^23,38,42^ and provides encouraging support for this mode of service delivery. The clinician log revealed no significant errors or difficulties using the iCILT software, suggesting that it is an appropriate tool for the delivery of CILT online to people with aphasia.

### Therapeutic effect

Improved confrontation naming, as measured by the CAT Naming subtest, was noted for both participants, with LR experiencing a clinically significant positive change. The improvement in naming performance for LR following iCILT is consistent with results from face-to-face CILT.^25,27,40^ The clinically significant improvement in written comprehension for LR was somewhat unexpected given that treatment did not target written language specifically. However, LR frequently requested the therapist to write the treatment targets that were difficult to produce due to his dyspraxia to facilitate successful production. This extra orthographic cueing may have contributed to his improvement at post-testing. It has also been proposed that the use of words in behaviorally relevant contexts, such as in requesting objects in iCILT, facilitates multimodal changes in brain reorganization.^41^ LR’s changes in written comprehension and improvements across all other subtests for either one or both participants is encouraging and may support this hypothesis. As few CILT studies utilize outcome measures that include assessment of written language, the results of this study and improvements noted by Meinzer and colleagues^28^ suggest that further investigation is warranted.

Although both participants demonstrated some improvement in language functioning, only BG showed improvement on the ALA, albeit to a minimal degree. Contention exists in the literature regarding the relationship between gains at the impairment level and change in activities, participation, and emotional wellbeing.^43^ Analysis of BG’s responses suggested selective improvements on questions related to aphasia severity and how much of a “problem” aphasia was for her. Less notable positive gains were reported in questions relating to participation in everyday life and personal factors, and an overall decrease was observed for questions relating to external factors such as the communication environment provided by others. Therefore, in this case, improvements on the ALA appeared to be more related to language functioning. LR’s results on all domains of the ALA decreased at post-treatment assessment which was an unexpected finding. It is well recognized that QOL is dynamic and influenced by a number of factors unable to be controlled for factors such as major life events, and personal factors (e.g. low psychological wellbeing).^4,44^ This may have been the case for LR who identified a number of other stressors in the home environment when completing the ALA at the post-assessment session. It is also possible that the two-week time frame between pre- and post- administration of the ALA may have influenced the results. Although test–retest reliability within this time period has been found to be good,^45^ few studies have used the ALA as an outcome measure for short-term interventions. In fact, the ALA has been administered up to six weeks after intensive interventions^46^ and may more genuinely reflect treatment-induced changes. Further research would assist in identifying the effects of iCILT on QOL for different aphasia profiles and over time.

It is important to note that neither participant received the full 30 h of therapy which may have negatively impacted treatment outcomes. Although network connection difficulties resulted in some loss of therapy time, participants asked to discontinue sessions on a number of occasions due to fatigue. Participant fatigue was also reportedly observed by the clinician in the session with participants demonstrating decreased accuracy in the production of target words, slower progression through the treatment hierarchy and increase in errors related to dyspraxia. This was particularly evident on day 4, 5, 9, and 10 of the treatment schedule. Fatigue is commonly reported post-stroke and is noted to negatively impact rehabilitation outcomes.^47^ Patient fatigue is reported by clinicians as impacting therapy participation in intensive treatments, particularly for older participants.^48^ In addition, computer based therapies may also exacerbate fatigue for some people with aphasia.^49^ The findings of this pilot study suggest that not all people with aphasia are able to tolerate high-intensity online intervention, and patient endurance may be a significant consideration for treatment candidacy.^50^

### Limitations and future directions

A number of limitations were identified in this study, most notably the connection issues experienced in Adobe Connect. Refinement of the videoconferencing software used should be explored for future studies. This is especially important for participants with limited computer skills and without support in the home, who are unable to troubleshoot or reconnect independently. Videoconferencing software running independently of a larger network may improve this limitation in future studies. It is, however, encouraging that no errors or difficulties were found with the iCILT card game developed by the investigators at either the participant or clinician end. Therefore this software program shows promise as an innovative and reliable clinical tool for the administration of CILT in the online environment.

It is acknowledged that this study has a small sample size with only two participants, and that the findings cannot be generalized to telerehabilitation treatment of the aphasia population. Further, participant characteristics may have influenced the results including severity of aphasia, co-occurring apraxia of speech and motivation to participate in intensive therapy. The participants also differed considerably in severity of language impairment. As suggested by Difrancesco and colleagues,^32^ future studies should consider matching each participant’s aphasia profile more closely to promote motivation and improve session dynamics. Despite these factors, the participants’ responses to therapy are similar to reported findings of face-to-face CILT. Considering the encouraging findings from this feasibility study, a larger study involving participants of varying aphasia types and severities is warranted.

## Conclusions

The aim of the study was to conduct a preliminary investigation into the feasibility of the delivery of high-intensity, evidenced-based CILT via telerehabilitation. Technical feasibility was established and participant satisfaction with the online treatment was high, suggesting that people with aphasia would be satisfied accessing this type of therapy in the online environment. Results from these two participants on language and communication-related QOL measures varied; however, the findings suggest that iCILT could yield a therapeutic effect in people with chronic aphasia. A number of benefits associated with the telerehabilitation treatment, most notably reduced travel and the ability to access high intensity services in the home, were identified. In the current healthcare environment where access to services is difficult for people with aphasia and the costs of services are high, the exploration of efficacious and efficient alternative service delivery models is essential. These findings provide support for further investigation of iCILT and other evidence-based aphasia treatments delivered via telerehabilitation.
